# Bioactive Modified Non-Wovens as a Novel Approach of Plants Protection against Invasive Slugs

**DOI:** 10.3390/ma14237403

**Published:** 2021-12-02

**Authors:** Marcin Rosowski, Dorota Puchowicz, Monika Jaskulska, Jan Kozłowski, Małgorzata Cieślak

**Affiliations:** 1 Department of Chemical Textiles Technologies, Lukasiewicz Research Network—Textile Research Institute, Brzezińska 5/15, 92-103 Łódź, Poland; marcin.rosowski@iw.lukasiewicz.gov.pl (M.R.); dorota.puchowicz@iw.lukasiewicz.gov.pl (D.P.); 2 Department of Entomology and Agricultural Pests, Institute of Plant Protection—National Research Institute, Władysława Węgorka 20, 60-318 Poznań, Poland; m.jaskulska@iorpib.poznan.pl (M.J.); jan.jacek.kozlowski@gmail.com (J.K.)

**Keywords:** bioactive non-wovens, slugs, pest control, crop protection, agrotextile, tansy flower

## Abstract

Invasive slugs generate significant problems in the area of horticultural and agricultural production. Despite the multitude of methods to reduce the pest population, including preventive, mechanical, agrotechnical, cultivation, biological, and chemical treatments, no effective plant protection strategy has been developed so far. In this paper, a solution based on modified non-woven fabric with bioactive molluscicidal properties using the extract of tansy flower, metaldehyde, and abamectin (Vertigo^®^ 018 EC) was proposed. All modified mats show significant anti-slug properties in comparison to control, and molluscicidal properties depend on the type of active substance. Non-woven modified with commonly used metaldehyde demonstrated fast action against slugs and presents the highest efficiency. The effectiveness of non-woven mats with Vertigo^®^ 018 EC is lower than for the mats with metaldehyde but higher than for the mats modified with tansy flower extract. The proposed solution will enable removing and neutralization of molluscicide from the fields, after the efficient pest control, according to circular economy principles. Moreover, it may allow for better control of the molluscicide release to the environment in comparison to widely used pellets, and contribute to the virtual protection of plants against invasive slugs.

## 1. Introduction

The increasing awareness of the need to protect the natural environment drives the search for novel solutions also in the field of agrotextiles and pest control. One of the significant problems for agrocenoses is the risk caused by terrestrial gastropods, which generates significant problems in the area of European horticultural and agricultural production [1,2,3]. It was calculated that the lack of effective slug control products may cost around GBP 100 million per year in lost crop production in the UK alone [4]. Among the slugs causing the greatest damage to crops are *Deroceras reticulatum* (Müller) and *Arion vulgaris* (Moquin-Tandon), *Arion rufus* (Linnaeus) [5]. *A. vulgaris* is an extremely invasive slug originating from forested habitats in the south-western part of Europe and *D. reticulatum* is a native slug in Europe with a worldwide distribution [6,7,8]. Protection of plantations and home gardens against terrestrial gastropods mainly consists of using various methods limiting their population and the plants’ damage. These include preventive, mechanical, agrotechnical, cultivation, biological, and chemical treatments. Preventive treatments consist of: drying; clearing ditches and shrubs; removing plant residues, stones, branches, and household waste. These activities limit the habitats of slugs and possible food sources. Agricultural techniques (procedures) are based on the mechanical destruction of pests by hoeing, harrowing, and tamping the soil in the field inter-rows. Another solution is the formation of barriers, e.g., in the form of sawdust, sand mixed with ash, or small fences, belts from copper foil [1]. Traps, raking, weed removal, and usage of mineral fertilizers may also help fight the pest. One of the recent eco-friendly solutions is the usage of fermenting bread dough as cheap and effective attractant for slugs [9]. An important solution is the use of the parasitic nematode e.g., *Phasmarhabditis hermaphrodita* (Schneider), *Phasmarhabditis neopapilliosa* (Mengert in Osche, Andrássy), *Phasmarhabditis tawfiki* (Azzam). Nematodes actively search for terrestrial gastropods–which are their hosts–and multiply in them, which leads to the significant feeding inhibition or death of the slugs [9]. Moreover, gastropod-killing beetles (C*arabidae*) may be an alternative biological control method of slugs in agricultural fields. They are particularly important predators of slugs and common in agrocenoses [10,11]. Moreover, various natural and synthetic chemical compounds and mixtures are widely used to protect plants against invasive slugs. These substances may act as a deterrent or an antifeedant which reduce the taste of plants and limit slugs’ feeding on young crops or act as a poison which causes dehydration of the body or digestive failure and death [2,12]. Repellent plants are also used, such as: thyme (*Thymus vulgaris* L.), marjoram (*Origanum majorana* L.), white mustard (*Sinapis alba* L.), salvia (*Salvia* L.), savory (*Satureja hortensis* L.), bouncing bet (*Saponaria officinalis* L.), wormwood (*Artemisia absinthium* L.), common bracken (*Pteridium aquilinum (L.) Kuhn*) etc. [12,13,14]. One of the most popular synthetic active substance in the anti-slug pellet is metaldehyde [15]. It has been widely used as a molluscicide since the ‘40 s [16,17]. Unfortunately, during rainfalls, these substances are washed away from fields and are released into the environment in an uncontrolled way, especially into the soil than to hydrosphere–where they are responsible for contamination of, e.g., drinking water [18,19,20,21]. Additionally, molluscicides have harmful effects on animals, including pets [22,23]. Therefore, an important issue is to design new forms of plant protection against slugs. The aim of the study was to design non-wovens modified with natural bioactive agent and commonly used agents as a comparison. Devised solution may constitute a novel approach in protection of plants against invasive slugs. A method of applying tansy flower extract (*Tanacetum vulgare* L.) and pesticides (abamectin and metaldehyde) to non-woven fabric was developed and the effectiveness of non-woven mats was tested, determining their influence on the condition and vitality of slugs. The proposed solution is innovative in the area of pest management, environmentally friendly, safer for non-target animals including pets due to usage of natural substances, and their binding to non-woven. Moreover, it may contribute to the virtual protection of plants against pests and enable better control of pesticide release into the environment. Furthermore, replacing of chemicals with the bio-based active agent in the finishing process and usage of recycled fibres may decrease negative impact on the environment. The binding of active bio-molluscicide with the non-wovens may allow for effective pest management and possible further re-use or recycling of modified agrotextile according to circular economy principles.

## 2. Materials and Methods

### 2.1. Materials

As a material for bioactive modification, a polypropylene (PP) non-woven was used (Table 1). The aqueous dispersion of a thermoplastic acrylic copolymer-based on acrylic-acid ester and vinyl ester Acronal^®^ 500 D was purchased from BASF (Ludwigshafen, Germany). A commercially available pesticide with abamectin 18 g/L as an active compound Vertigo^®^ 018 EC was delivered by ADAMA Polska (Warszawa, Poland). Metaldehyde from Sigma–Aldrich (Steinheim, Germany) was used as a modifier and as a standard molluscicide.

**Figure 1:**
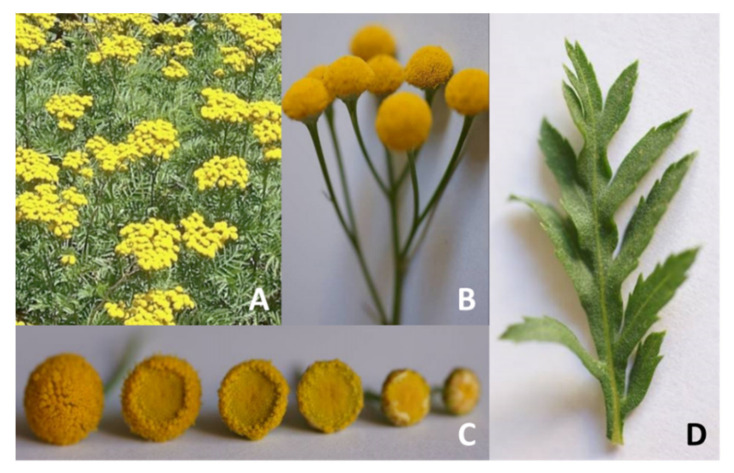
Tansy: (**A**) overview, (**B**) button-like flower, (**C**) different state of tansy anthodium, (**D**) finely divided compound leaf.

Ethyl alcohol 98% for extraction ordered from Chempur (Piekary Śląskie, Poland) and Linseed oil from Oleofarm (Wrocław, Poland). Tansy (*Tanacetum vulgare* L.) family *Asteraceae* Figure 1—part of the inflorescence was picked from the anthodium of tansy. The collection was carried out during the flowering period in Łódź. The flowers were dried in the dark at ≤20 °C.

For the anti-slug activity test, a culture of widely spread grey field slug (*D). reticulatum*) was chosen. The grey field slug occurs throughout Poland and is quite commonly found in crop fields and gardens. It is the most serious slug pest of winter oilseed rape and winter wheat as well as brassicas grown in gardens and fields. The grey field slug is up to 4.5 cm long and creamy or light coffee, with brownish or blackish spots. Its maximum age is about 12 months [24].

### 2.2. Methods

#### 2.2.1. Preparation of Active Dispersion 

Tansy flowers were poured with ethyl alcohol (1:4 *w/w*), tightly closed and left in the dark at 20 °C for 74 h. After this time, the extract was decanted, and linseed oil was added (1:4 *v/v*) and then the mixture was shaken. The obtained mixture was added to Acronal^®^ 500 D to obtain active dispersion with 7% (*w/w*) tansy flower oil extract. Active dispersion with metaldehyde and Vertigo^®^ 018 EC was prepared by mixing with Acronal^®^ 500 D to obtain two mixtures with the same concentration of 7% (*w/w*). The polymer dispersion with the addition of bioactive modifiers was stirred using a mechanical laboratory stirrer for 10 min. at a rotation speed of 25 rpm [25].

#### 2.2.2. Preparation of Modified Non-Woven Mats

The modified polymer dispersions were applied onto non-woven samples (25 cm × 25 cm) and spread using a metal roller (0.3 kg). The samples were heated for 10 min at 80 °C in the laboratory drier. Sample names are listed in Table 2.

#### 2.2.3. Scanning Electron Microscopy (SEM)

Images of the non-woven mats were collected using scanning electron microscope Vega 3 SEM (Tescan, Brno, Czech Republic) equipped with a secondary electron detector (SE) and backscattered electron detector (BSE). To visualize the entire surface the magnifications of 50× and 1000× were selected. The prepared sample was placed onto an aluminum specimen mount with carbon tape; after that, a thin gold layer was sputtered onto the sample surface using Q150R ES coater (Quorum, Laughton, UK) to prevent charging of the specimen. All measurements were performed using ImageJ software from National Institutes of Health (v.1.8.0_172, Bethesda, MD, USA).

#### 2.2.4. Raman Spectroscopy

Raman spectroscopy studies were performed using a Renishaw InVia Reflex dispersive spectrometer with Leica microscope (Renishaw, Wotton under Edge, UK). An excitation source of λ = 785 nm, 300 mW was applied with spectral resolution 1 cm^−1^. Laser power was dependent on the sample and varied from 1% to 10% of the power. The spectra were accumulated within 10–300 s integration time. Test conditions T = 22 ± 2 °C, RH = 40 ± 2%. The analysis by Raman technique was carried out in a closed microscope chamber of the spectroscope with samples being placed on the microscope plate. Samples were positioned in the laser light focus using a microscope (magnification 50×) with CCD camera. The recording of spectra was carried out by Renishaw WiRE™ software (v.3.2, Wotton under Edge, UK). All Spectra processing was done with the use of Origin software (OriginLab, v.8.0, Northampton, MA, USA).

#### 2.2.5. *D. reticulatum* Breeding

The population of *D. reticulatum* was obtained several times a year from a town in the vicinity of Poznań. The captured slugs were kept in a 5 cm layer of soil in plastic containers at 16–17 °C. They were fed on cabbage leaves, potato tubers, carrot roots, and wheat bran with the addition of calcium carbonate. Food was changed every three days. The average weight of used slugs was around 0.391 g.

#### 2.2.6. ‘Hilton’ Napa Cabbage Breeding

Plants of ‘Hilton’ Napa cabbage (*Brassica pekinensis* (Lour.) Rupr.) cultivar were obtained from seeds grown in raised beds in the greenhouse of the Institute of Plant Protection, National Research Institute in Poznań. Cabbage plants in a stage of 5–6 leaves were obtained after 8 weeks of growth.

#### 2.2.7. Anti-Slug Activity Test

**Figure 2:**
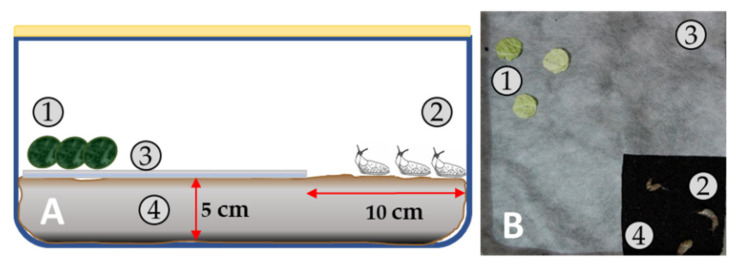
Experimental setup for testing an anti-slug activity of modified mats: (**A**) scheme, (**B**) top view image. Components: (**1**) Nappa cabbage discs φ 2.5 cm, (**2**) *D. reticulatum* slugs, (**3**) Modified non-woven mat, (**4**) Soil layer.

The tests were carried out in laboratory conditions at 16 ± 1 °C, RH = 93% ± 2% and a day length of 12 h. As a feed, the disk of Napa cabbage with a diameter of 2.5 cm was used. In plastic containers, non-woven mats (25.0 cm × 25.0 cm) modified with the following active emulsion (Table 2) were placed on the 5 cm soil layer. In each container, three slugs (*D. reticulatum*). were placed on the free surface of the soil (10.0 cm× 10.0 cm) and a disk of Napa cabbage (3 per container) were allocated into the container (Figure 2). The condition and vitality of slugs (healthy, sick and dead) and the size of plant damage were assessed daily according to a five-point scale (0—no damage, 25, 50, 75 and 100% of damaged cabbage disk surface). For each sample (modified non-woven mats and control samples) six repetitions were performed. The obtained results were statistically analyzed using Tukey’s test at the significance level of α = 0.05.

## 3. Results and Discussion

### 3.1. Scanning Electron Microscopy (SEM)

**Figure 3:**
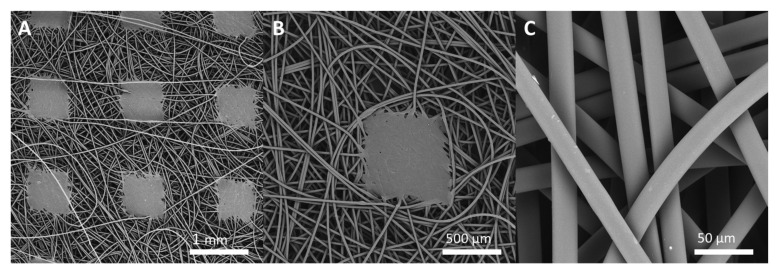
SEM images of: (**A**–**C**) non-woven polypropylene mat. All images were collected at accelerating voltage 20 kV using BSE detector. Magnification: (**A**) 10×, (**B**) 50×, (**C**) 1000×.

To characterize the surface of the non-woven scanning electron microscopy was used. SEM images of the nontreated mat (Ctrl_PP) is presented in Figure 3A–C. Fibre diameter was determined based on a measurement of at least ten fibres from the randomly selected SEM images. The results clearly indicate that the fibres are uniform with a size of 20.4 ± 0.9 μm. Moreover, the surface of the individual calendar square pattern was estimated to be about 0.51 ± 0.02 mm^2^ and the pattern percentage about 15%/1 m^2^.

### 3.2. Raman Spectroscopy

**Figure 4:**
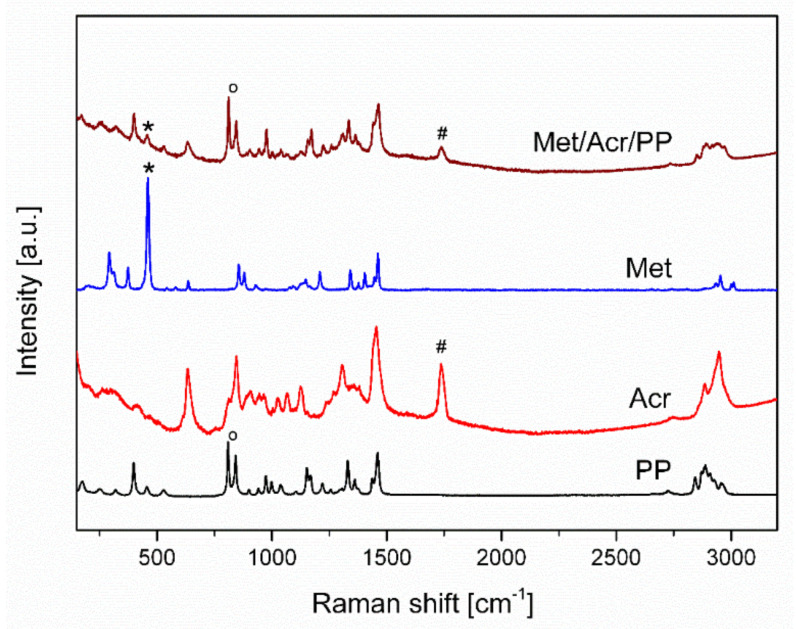
Raman spectra of PP mat modified with Acronal^®^ 500 D and metaldehyde and spectra of all constituents. ^O^—peak of CH_2_ wagging, CCH_3_ stretching (809 cm^−1^) and CC backbone stretching, CH_2_ wagging, CCH_3_ stretching and CH_3_ bending (847 cm^−1^) of PP, #—peak of the C=O stretching of Acr, *—peak for deformation vibration of epoxy compounds of Met. Samples acquisition parameters: power/integration time: PP: 1%/10 s; Acr: 1%/300 s; Met: 1%/10 s; Met/Acr/PP: 1%/120 s.

For the characterization of modified non-woven mats, Raman spectroscopy was used. In the Raman spectrum of metaldehyde (Met) the most intense band occurs at 460 cm^−^^1^. This is a characteristic peak for the deformation vibration of epoxy compounds (COCOC) [26]. It can be also recognized on the spectrum of modified PP non-woven (Figure 4).

**Figure 5:**
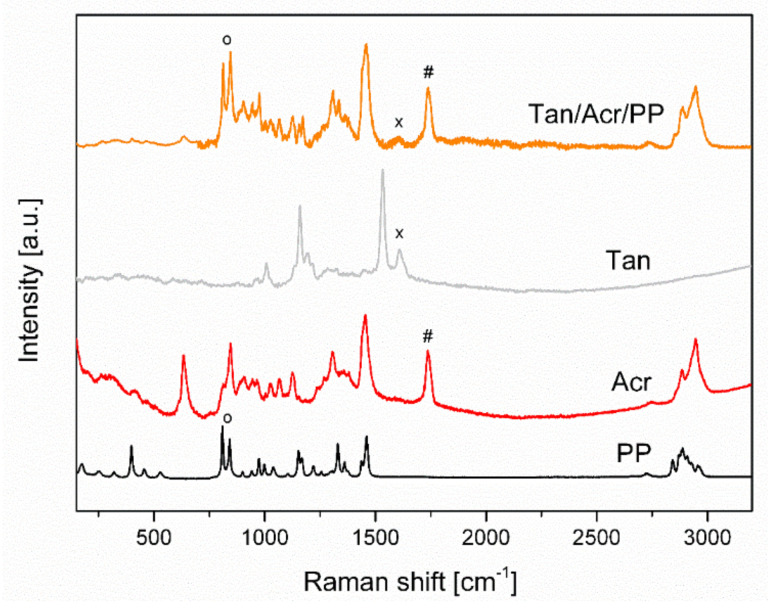
Raman spectra of PP mat modified with Acronal^®^ 500 D and Tansy flowers extract and spectra of all constituents. ^O^—peak of CH_2_ wagging, CCH_3_ stretching (809 cm^−1^) and CC backbone stretching, CH_2_ wagging, CCH_3_ stretching and CH_3_ bending (847 cm^−1^) of PP, #—peak of the C=O stretching of Acr, x—region of the N-H and O-H bending vibrations. Samples acquisition parameters: power/integration time: PP: 1%/10 s; Acr: 1%/300 s; Tan: 1%/10 s; Tan/Acr/PP: 1%/120 s.

**Figure 6:**
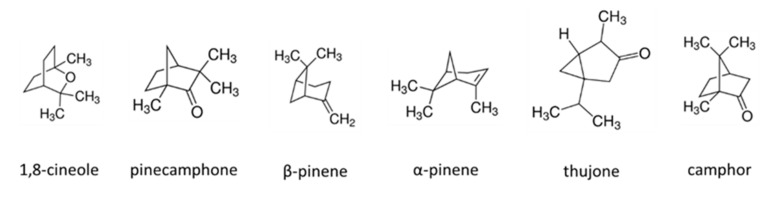
The structural formula of main active compounds of tansy.

Acronal^®^ 500 D is a dispersion of an acrylate copolymer with a characteristic band at 1738 cm^−^^1^ which is the result of the C=O stretching (Acr). For the PP mat two bands are characteristic. They are assigned to CH_2_ wagging, CCH_3_ stretching (809 cm^−^^1^) and CC backbone stretching, CH_2_ wagging, CCH_3_ stretching and CH_3_ bending (847 cm^−^^1^) [27] (Figure 4). On modified non-woven mat with metaldehyde (Met/Acr/PP) characteristic bands of Acronal^®^ 500 D at 1738 cm^−^^1^ and metaldehyde at 460 cm^−^^1^ are detected and confirm the modification. The most intense bands are at 1532 cm^−^^1^ and 1612 cm^−^^1^ in the region of the N-H and O-H bending vibrations. On non-woven modified mat Tan/Acr/PP characteristic bands of Acronal^®^ 500 at 1738 cm^−^^1^ and weak band of tansy at 1532 cm^−^^1^ was detected which corresponds to C=C stretching of e.g., pinene (Figure 5). Tansy flower constitutes from more than 200 compounds [28] and its chemical composition is not the same everywhere. It depends on the geographic region [29]. The structural formulas of main compounds are shown in Figure 6.

**Figure 7:**
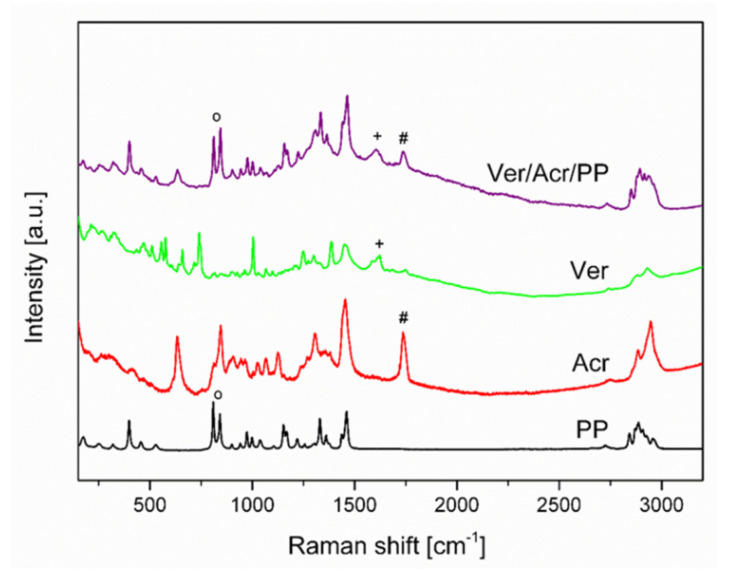
Raman spectra of PP mat modified with Acronal^®^ 500 D and Vertigo^®^ 018 EC and spectra of all constituents. ^O^—peak of CH_2_ wagging, CCH_3_ stretching (809 cm^−1^) and CC backbone stretching, CH_2_ wagging, CCH_3_ stretching and CH_3_ bending (847 cm^−1^) of PP, #—peak of the C=O stretching of Acr, +—characteristic band for the Vertigo^®^ 018 EC. Samples acquisition parameters: power/integration time: PP: 1%/10 s; Acr: 1%/300 s; Ver: 10%/90 s; Ver/Acr/PP: 1%/60 s.

In Raman spectrum of commercially available product Vertigo^®^ 018 EC based on abamectin, the characteristic intense band occurs at 1621 cm^−^^1^ (ring bending and C=C stretching of abamectin). This band is also recognizable in Ver/Acr/PP spectrum (Figure 7). Abamectin, a natural product from the group of macrocyclic lactones, is a substance isolated in the fermentation process from the soil bacterium *Streptomyces avermitilis* [30]. The characteristic carbonyl band of the lactone occurs around 1747 cm^−^^1^.

In Table 3 the maxima of characteristic bands of active compounds (modifiers) were listed.

Based on the results from the Raman technique, the presence of active modifiers on the non-woven surface was confirmed. They can be distinguished among the characteristic peaks of PP and Acronal^®^ 500 D.

### 3.3. Anti-Slug Activity Test

**Figure 8:**
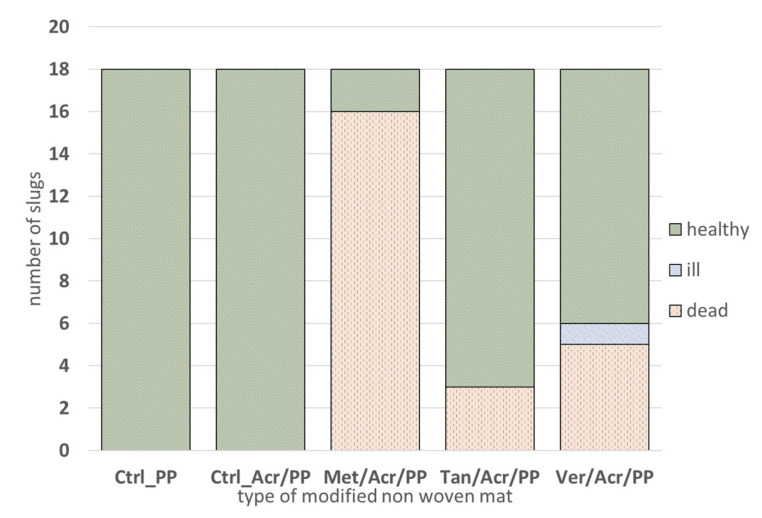
Number of healthy, ill, and dead *D. reticulatum* after seven days of observation on control and modified non-woven mats. Tukey’s test was conducted at a significance level of α = 0.05.

During the test, the vitality of *D. reticulatum* and the damage extent of Napa cabbage discs were evaluated. The lethal effect of active substances (modifier) after seven days of experiment on *D. reticulatu*m with the modified non-woven mat is shown in Figure 8.

The first dead slugs were found after two days of the experiment. There were 6 dead individuals for metaldehyde modified mats and one for tansy extract modified mats. On the third day of the experiment, the number of dead slugs was 11 for Met/Acr/PP, 0 for the Tan/Acr/PP, and 2 for Ver/Acr/PP. In the following days, a few more dead slugs were recorded for all three modified non-woven mats. After seven days of the experiment, the highest mortality of slugs (16 molluscs) was found for Met/Acr/PP mats, 5 slugs for Ver/Acr/PP mats and 3 for Tan/Acr/PP mats. During the entire experiment, no dead or ill slugs (the slugs did not feed, move and were not interested in food and have glassy altered skin surface) were recorded for the control mats (with and without Acronal^®^ 500 D). Finale statistical analysis of Tukey’s test concerning the number of healthy slugs during seven days of the experiment showed that Met/Acr/PP was significantly different from the other versions (controls and modified non-woven mats) and Ver/Acr/PP was significantly different from the controls (Ctrl_PP, Ctrl_Acr/PP) and Met/Acr/PP but was not significantly different from Tan/Acr/PP.

After 24 h of the experiment, significant differences in the damage extent of the Napa cabbage discs by *D. reticulatum* were observed (Table 4). The cabbage discs placed on modified mats were less damaged in comparison to the controls (Ctrl_PP, Ctrl_Acr/PP). From the third day to the end of the observation, the cabbage discs in the containers with the control mats were significantly more nibbled than the disks placed on the mats modified by active substances. From the first to the last day of the experiment, the damage extent of the discs placed on the Met/Acr/PP was constant and was estimated to be about 2.8%. After seven days, the damage extent of the cabbage discs on the metaldehyde, tansy extract, and Vertigo^®^ 018 EC modified mats was 2.8%, 38.9%, and 43.1%, respectively, while the discs on the control mats without and with Acronal^®^ 500 D showed the damage around 80.6% and 56.9% respectively. The combined analysis of the damage degree during seven days of the slugs feeding showed that mats containing metaldehyde, tansy extract and Vertigo^®^ 018 EC had a significant impact on reducing the damage extent of Napa cabbage discs by *D. reticulatum*. No phytotoxicity of modified mats was observed on the Napa cabbage discs upon visual inspection.

## 4. Conclusions and Summary

A modern approach of application of natural extract of tansy flower and commercially available pesticides (metaldehyde and abamectin) to PP non-woven was elaborated and the effectiveness of modified non-woven mats was tested. Raman spectroscopy results confirmed presence of bioactive substances on the non-woven mats. Based on the results of anti-slug test it was concluded that molluscicidal properties depend on the type of active substance. Non-wovens modified with commonly used metaldehyde demonstrated fast action against slugs and presents the highest efficiency. For this system, further studies on the possibility of reducing metaldehyde concentration should be conducted. The mats modified with tansy flower extract exhibited slower action against slug, but a significant advantage is the fact that tansy flower extract is of natural origin. According to literature, tansy contains a rich mixture of saturated and unsaturated cyclic compounds. Camphor, umbelloni, borneol, α-and β-thujone, 1,8-cineol, sabinene and α- and β-pinene are among the more important. Moreover, sesquiterpene lactones e.g., tanacetin, arbosculin, and flavonoids such as quercetin, diosmin and apigenin are present [28,29,31,32,33]. Camphor, 1,8-cineol demonstrates proven and pronounced toxic effect on sand hill snail [34]. Additionally, α- and β-thujone exhibit acaricidal; insecticidal; larvicidal; pesticidal; and insect repellent properties [35,36]. These bioactive substances are responsible for molluscicidal activity in Tan/Acr/PP mats. The effectiveness of non-woven mats with Vertigo^®^ 018 EC is lower than for the mats with metaldehyde but higher than for the mats modified with tansy flower extract. Moreover, it has been proven in the literature that treating slugs’ fodder or plants with Vertigo^®^ 018 EC significantly reduces plant damage. Abamectin act as deterrent and antifeedant to slugs [37]. All modified mats show significant anti-slug properties in comparison to pure PP mat. Novel approach of plants protection proposed in this paper may be used on non-woven fabrics made from different: raw material type, composition, and structure, depending on the product confectioning. An important issue is an application of the active compounds (e.g., natural bioactive extracts, pesticide etc.) to the non-woven fabric. This solution may allow for better control of the molluscicide release to the environment in comparison to widely used metaldehyde pellet. The binding of active substances to the non-wovens will enable their removing and neutralization from the fields according to circular economy principles after the efficient pest control.

## Figures and Tables

**Table 1:** Characteristic of non-woven mat.

Type	Weight [g/m^2^]	Thickness [mm]
Polypropylene (PP), spun bonded	35.0 ± 5	0.46 ± 0.1

**Table 2:** List of sample names.

Sample Name	Sample Composition
Ctrl_PP	Control: PP mat
Ctrl_Acr/PP	Control: PP mat + Acronal^®^ 500 D (100%)
Met/Acr/PP	PP mat + Acronal^®^ 500 D (93%) + metaldehyde (7%)
Tan/Acr/PP	PP mat + Acronal^®^ 500 D (93%) + tansy flowers extract (7%)
Ver/Acr/PP	PP mat + Acronal^®^ 500 D (93%) + Vertigo^®^ 018 EC (7%)

**Table 3:** The maxima of Raman characteristic bands of modifiers.

Raman Bands [cm^−1^]		
Metaldehyde	Tansy Flowers Extract	Vertigo^®^ 018 EC
293		214
374		324
460		510
636		576
856		658
880	722	740
1148	1010	1004
1209	1160	1247
1341	1195	1386
1404	1532	1455
1462	1612	1621
		1747

**Table 4:** The influence of modified non-woven mats on the corrected average damage extent of Napa cabbage discs (average in %), by *D. reticulatum* and the results of Tukey’s test at the significance level of α = 0.05. a–c—values in columns marked with the same letters do not differ significantly.

Sample Name	Days of Feeding						
	1	2	3	4	5	6	7
Ctrl_PP	27.8 b	48.6 b	59.7 c	75.0 c	77.8 c	79.2 c	80.6 c
Ctrl_Acr/PP	5.6 a	16.7 a	26.4 b	33.3 b	47.2 b	52.8 b	56.9 bc
Met/Acr/PP	2.8 a	2.8 a	2.8 a	2.8 a	2.8 a	2.8 a	2.8 a
Tan/Acr/PP	1.4 a	4.2 a	9.7 ab	18.1 ab	31.9 b	36.1 b	38.9 b
Ver/Acr/PP	0.0 a	1.4 a	5.6 a	19.4 ab	27.8 ab	36.1 b	43.1 b

## Data Availability

The data that support the findings of this study are available on request from the corresponding author.
